# Real-world experiences: Efficacy and tolerability of pirfenidone in clinical practice

**DOI:** 10.1371/journal.pone.0228390

**Published:** 2020-01-30

**Authors:** Chuling Fang, Hui Huang, Jian Guo, Martin Ferianc, Zuojun Xu

**Affiliations:** 1 Department of Respiratory Medicine, Peking Union Medical College Hospital, Chinese Academy of Medical Sciences & Peking Union Medical College, Beijing, China; 2 Electronic and Electrical Engineering Department, University College London, London, United Kingdom; Medical Center - University of Freiburg, GERMANY

## Abstract

**Background:**

The safety of pirfenidone on pulmonary fibrosis patients with other kinds of interstitial lung diseases (ILDs) in addition to idiopathic pulmonary fibrosis (IPF) is unknown. Furthermore, its effectiveness-related factors on IPF patients are not quite explored.

**Methods:**

A retrospective study, on patients prescribed pirfenidone for pulmonary fibrosis, was conducted to assess effectiveness on IPF patients and tolerability of all patients with lung fibrosis. The effectiveness of pirfenidone was tested on 110 IPF subjects receiving treatment for ≥3 months by high-resolution computed tomography (HRCT). Response-linked factors and progression-free survival (PFS) were also analyzed. The data about safety outcomes and drug dose adjustments were collected from all included subjects.

**Results:**

A total of 176 subjects were included: 117 were IPF, 19 connective tissue disease-associated interstitial lung disease (CTD-ILD), and 40 unclassifiable ILD. Out of the 110 IPF subjects, 89 subjects were assessed as stable and 21 as progressive, out of which 10 died of acute exacerbation and 11 progressed. The effectiveness was significantly related to their baseline body mass index (BMI). IPF subjects with BMI>25kg/m^2^ or diffusion capacity of carbon monoxide (DLco)>30% had higher PFS rate. The most common adverse events were skin-related and gastrointestinal-related. Drug discontinuation owing to adverse events occurred similarly in these three groups.

**Conclusion:**

Pirfenidone was well tolerated in most of the lung fibrosis patients besides IPF, with a similar pattern of adverse events. Nearly 80% of IPF subjects were assessed as stable. More benefits were seen in IPF patients with higher BMI or mild-to-moderate disease.

## Introduction

Idiopathic pulmonary fibrosis (IPF) is a chronic, progressive and life-threatening fibrotic lung disease characterized by the irreversible decline in lung function, with an estimated median survival of 2–3 years. Although IPF has an overall poor survival rate, the natural clinical course of individual patient varies from slow progression to acute exacerbation and death[1]. Current treatment strategies advocate nonpharmacologic management and pharmacologic therapy[2]. The former one contains smoking cessation, supplemental oxygen[3], pulmonary rehabilitation[4] and lung transplantation[5]. The latter one mainly refers to anti-fibrotic therapies, with pirfenidone and nintedanib being shown to be effective and safe in the treatment of IPF[6].

Pirfenidone (5-methyl-1-phenyl-2-[1H]-pyridone), a bioavailable synthetic molecule, is the first drug to be approved for use on IPF patients. It is an anti-inflammatory and anti-fibrotic agent that down-regulates transforming growth factor beta (TGF-β) and tumor necrosis factor alpha (TNF-α), inhibits collagen synthesis and reduce fibroblast proliferation[7,8]. Two largest randomized clinical trials for pirfenidone on IPF patients, the CAPACITY and ASCEND trials, demonstrated a slower decline of the lung function and sufficient drug tolerability[9,10]. These two clinical trials and the long-term follow-up of the subjects showed that the most common adverse events were skin-related like rash and gastrointestinal-related containing nausea and dyspepsia[9–11]. Research has been conducted on the use of pirfenidone in other types of pulmonary fibrosis, such as scleroderma-associated interstitial lung disease[12–14] and clinically amyopathic dermatomyositis[15], and potential benefits were reported.

Randomized controlled trials have been considered the gold standard for clinical evidence for a long time. However, in our real-world clinical practice, many patients who receive pirfenidone treatment have obvious medical comorbidities, concurrent medicines or poorer pulmonary function tests (PFTs) (FVC<50%, DLco<30% or 35% predicted) that would have excluded them from clinical trial participation. Additionally, clinical trials only included patients with IPF, and other types of interstitial fibrosis like CTD-ILD and unclassifiable ILD were excluded. Nevertheless, in our real-world clinical practice, patients with various kinds of lung fibrosis are going to receive pirfenidone therapy and the safety of pirfenidone on these patients are unclear. In this study, we explore the real-world experience of pirfenidone on patients with fibrotic ILDs besides IPF during an extended four years and two months periods.

## Methods

### Patients (inclusion and exclusion criterion)

We conducted a retrospective single-center research on 176 subjects prescribed pirfenidone for pulmonary fibrosis of any etiology from July 2014 to August 2018 in Peking Union Medical College Hospital (PUMCH), Dongcheng District, China. Inclusion criteria were as follows: (1) adult patients (≥ 18 years old) with the diagnosis of pulmonary fibrosis as determined by two experienced pulmonologists and one radiologist during patients’ routine clinical care. Pulmonary fibrosis patients with any etiology were enrolled, involving IPF, CTD-ILD and unclassifiable ILD. The diagnosis of IPF was in accordance with American thoracic society/European respiratory society (ATS/ERS) guidelines published in 2011[3]. The diagnosis of unclassifiable ILD was based on the consensus in 2002[16]/2013[17] and the review[18] in 2018. Patients in our research was diagnosed with unclassifiable ILD due to unclassified clinical/radiological condition and lack of biopsy. (2) Patients were treated with pirfenidone. Patients with other types of fibrotic lung diseases agreed to receive pirfenidone therapy after detailed consultation with their pulmonologists since the fibrosis was not controlled or in a progressive state despite adequate treatment with sufficient prednisone and immunosuppressive agents. In clinical practice, patients received pirfenidone at a starting dose of 300 mg/day (1 capsule, 3× daily) titrated to a maintenance dose of 1800 mg/day (6 capsules, 3× daily). Subjects were excluded if comorbidities including malignancy, severe hepatic dysfunction or renal disease occurred before treatment.

### Study design

This was a single center real-world retrospective study, which was approved by the Regional Ethics Committee of our hospital (JS-1127/2016). Due to the retrospective nature of the study, informed consent was waived.

### Baseline data

Subjects who met study inclusion criteria were continuously enrolled for analysis. The paper and electronic medical records of our hospital healthcare system were used to collect baseline data involving demographic information, smoking status, home oxygen therapy, comorbidities, concurrent medication, laboratory examination, PFTs, HRCT, lung biopsy results and prescribing information for pirfenidone therapy.

### HRCT score system

The HRCT scoring was independently performed by two experienced radiologists based on a semiquantitative visual assessment. The lung was divided into three zones (upper, middle and lower), each zone was evaluated separately. The upper lung zone was defined as the area of the lung above the level of the tracheal carina, the lower lung zone was the area of the lung below the level of the inferior pulmonary vein, and the middle lung zone was the area of the lung between the upper and lower zones. The extent of lung involvement was evaluated based on the percentage of lung parenchyma occupied by the lung fibrosis (reticular opacity, traction bronchiectasis and honeycombing) of each zone and then the average value was accepted. Subsequently, the lung fibrosis percentage of HRCT findings were graded on a scale of 1 to 4 as follows: areas with (1) <25% lung fibrosis, (2) 25~50% lung fibrosis, (3) 50~75% lung fibrosis and (4) >75% lung fibrosis[19].

### Effectiveness and safety outcomes

The effectiveness of pirfenidone was assessed on IPF patients receiving pirfenidone therapy for ≥ 3 months by HRCT. In our study, disease progression was defined as an occurrence of any one of the following: (1) a confirmed progression of fibrosis defined by the HRCT fibrosis score (with an increase of at least 1 point), (2) acute exacerbation of IPF (AE-IPF), or (3) death. AE-IPF definition in our study was in accordance with the American Thoracic Society/European Respiratory Society consensus criteria of IPF[16]. Response-linked factors and PFS were also analyzed. The PFS period was defined as the time until the first occurrence of progression mentioned above. In the daily clinical practice, most patients received PFTs in different hospitals and some might not have PFTs after pirfenidone therapy. Only 41 out of 110 IPF patients had PFTs before and after treatment in our hospital. In addition, because of the limited sample of the other two types of ILDs (CTD-ILD and unclassifiable ILD), the effectiveness of pirfenidone on them was not assessed in this research. Only acute exacerbation/death and time of these events were recorded.

Safety outcomes for the study included adverse events, drug dose adjustments, drug discontinuation because of an adverse event and time to drug discontinuation.

### Statistical analysis

Statistical analysis was performed using SPSS software version 19.0 for Windows (SPSS Inc., Chicago, IL). Two-tailed *P*<0.05 was considered statistically significant. Results of continuous variables were reported as mean ± standard deviation (SD). Statistical analysis was performed using Student’s t-test between groups of two measurement data which fulfilled homogeneity of variance. For those which did not conform to the homogeneity of variance, calibration t-test was used. Categorical variables were reported as a number with percentage and chi-square test was used to compare no less than 2 groups of categorical variables and Fisher’s exact test was used appropriately. A logistic regression model was performed to identify the factors linked to the effectiveness and withdrawal of pirfenidone. For time-to-event analysis, PFS time was compared between two groups with different BMI, smoking status and DLco predicted using a log-rank test; hazard ratios were based on the Cox proportional-hazards model.

## Results

### Baseline characteristics

**Fig 1:**
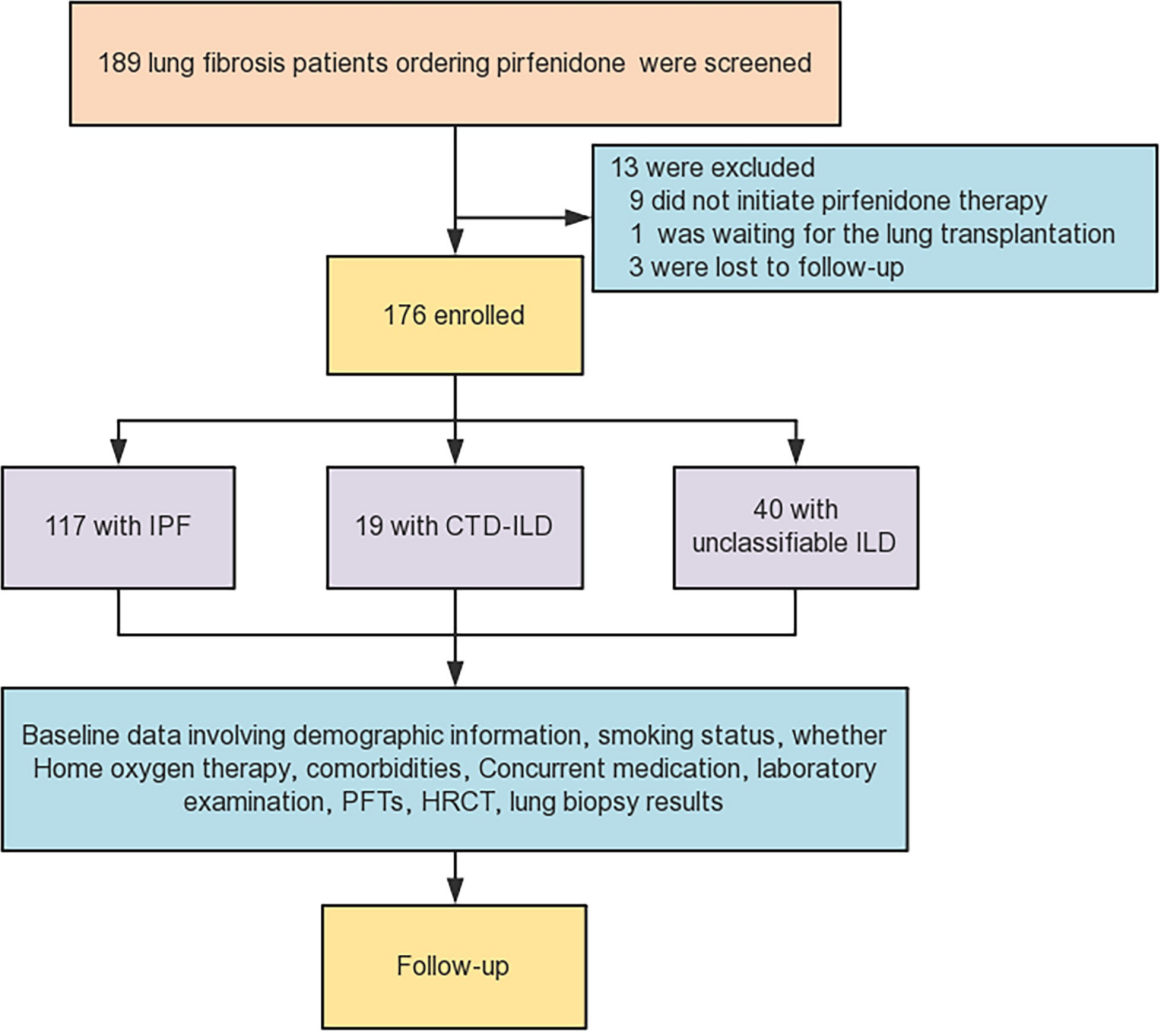
Flow diagram of the assessment of subjects included in the study. IPF = Idiopathic pulmonary fibrosis; CTD-ILD = connective tissue disease-associated interstitial lung disease; ILD = interstitial lung disease; PFTs = pulmonary function tests; HRCT = high-resolution computed tomography.

The procedure for identifying eligible subjects was summarized in Fig 1. A total amount of 176 subjects from the 189 consistent with our study criteria were included in our analysis. 9 subjects did not initiate pirfenidone therapy after the prior prescription, 1 subject was waiting for the lung transplantation and 3 subjects were unable to reach for the follow-up. Of the 176 subjects included, 117 were IPF, 19 CTD-ILD and 40 unclassifiable ILD. The observation period was 52±35 weeks in the IPF group, 50±25 weeks in the CTD-ILD group and 41±25 weeks in the unclassifiable ILD group. A significantly higher proportion of male subjects were found in the IPF group (94.0%) than in the other two groups (42.1% and 52.5% respectively). 61.5% of subjects in the IPF group were smokers, while a lower rate was found in the CTD-ILD (15.8%) or unclassifiable ILD group (22.5%). Medical comorbidities with the highest frequency of the three groups were diabetes mellitus (15.4%, 26.3% and 2.5% respectively), coronary artery disease (19.7%, 10.5% and 5.0% respectively) and hypertension (17.9%, 10.5% and 2.5% respectively). Prednisone was the most common concurrent medication used in the CTD-ILD group (73.2%) and unclassifiable ILD (47.5%) group. More baseline characteristics of the included subjects were listed in Table 1.

**Table 1:** Baseline characteristics.

Baseline characteristics	IPF group (n = 117)	CTD-ILD (n = 19)	Unclassifiable ILD (n = 40)
Treatment time of pirfenidone (weeks)	52±35	50±25	41±25
Age (years)	63.6±8.4	61.9±10.9	59.4±8.6
Male, n (%)	110 (94.0)	8 (42.1)	21 (52.5)
BMI (kg/m^2^)	24.4±2.9	23.6±2.5	23.2±2.3
Time since IPF/ILD diagnosis (years)	2.6±1.9	3.1±2.1	2.0±1.1
Surgical lung biopsy (including video-assisted thoracoscopic surgery and cryobiopsy), n (%)	12 (10.2)	1 (5.3)	0
Smoking status, n (%)			
Former smoker	60 (51.3)	3 (15.8)	9 (22.5)
Never smoker	45 (38.5)	16 (74.2)	31 (77.5)
Active smoker	12 (10.2)	0	0
Supplemental oxygen, n (%)	48 (41.0)	6 (31.6)	14 (35.0)
Comorbidities, n (%)			
CPFE	20 (17.1)	1 (5.3)	1 (2.5)
Asthma	3 (2.6)	0	0
Diabetes mellitus	18 (15.4)	5 (26.3)	1 (2.5)
Hypertension	21 (17.9)	2 (10.5)	1 (2.5)
Atrial fibrillation	5 (4.3)	0	1 (2.5)
Coronary artery disease	23 (19.7)	2 (10.5)	2 (5.0)
Congestive heart failure	7 (6.0)	0	0
Concurrent medication use, n (%)			
Acid reflux medication	18 (15.4)	2 (10.5)	2 (5.0)
Prednisone	8 (6.8)	12 (73.2)	19 (47.5)
Cyclophosphamide	3 (2.6)	3 (15.8)	5 (12.5)
Azathioprine	1 (0.9)	3 (15.8)	1 (2.5)
Methotrexate	0	1 (5.3)	0
Tacrolimus	0	0	2 (5.0)

Measurement data were presented as mean±standard deviation and enumeration data were reported as a number with percentage. CTD-ILD group included patients with rheumatoid arthritis-ILD (RA-ILD), primary Sjogren's syndrome-ILD (PSS-ILD), systemic sclerosis-ILD (SSc-ILD) and other CTD-ILD. CTD-ILD = connective tissue disease-associated ILD; ILD = interstitial lung disease; IPF = idiopathic pulmonary fibrosis; CPFE = combined pulmonary fibrosis and emphysema; GERD = gastroesophageal reflux disease; SD = standard deviation.

117 IPF patients (100%) were showing definite or possible UIP on HRCT, while only 47.4% of subjects with CTD-ILD and 10% with unclassifiable ILD exhibited UIP on HRCT. The remaining subjects had imaging features inconsistent with UIP pattern. The subjects in our research had more advanced pulmonary fibrosis as demonstrated by the large proportion of patients with HRCT scores of 2 or 3 or 4 (60.7%, 57.9% and 55% in three groups respectively) or those who required supplemental oxygen (41.0%, 31.6% and 35% respectively), and lower DLco predicted (45.1±13.6%, 48.6±14.0% and 50.0±16.0% respectively). The average of FVC predicted in all three groups was around 70–75%. More details were revealed in the Table 2.

**Table 2:** Baseline HRCT and PFTs.

Baseline characteristics	IPF (n = 117)	CTD-ILD (n = 19)	Unclassifiable ILD(n = 40)
HRCT pattern, n (%)			
Definite UIP pattern	106 (90.6)	3 (15.8)	1 (2.5)
Possible UIP pattern	11 (9.4)	6 (31.6)	3 (7.5)
Inconsistent with UIP pattern (including indeterminate UIP and consistent with non-IPF diagnosis)	0	10 (52.6)	35 (87.5)
No HRCT available for review	0	0	1 (2.5)
HRCT score			
1	46 (39.3)	8 (42.1)	18 (45.0)
2	56 (47.8)	10 (52.6)	16 (40.0)
3	14 (12.0)	1 (5.3)	5 (12.5)
4	1 (0.9)	0	1 (2.5)
PFT			
FVC (% predicted)	70.7±14.3	74.9±18.6	73.2±19.1
TLC (% predicted)	65.8±11.0	67.5±12.8	67.2±15.5
DLco (% predicted)	45.1±13.6	48.6±14.0	50.0±16.0

UIP = usual interstitial pneumonia; PFT = pulmonary function test; FVC = forced vital capacity; TLC = total lung capacity; DLco = diffusion capacity of carbon monoxide.

### Effectiveness analysis in subgroups of IPF patients

#### Effectiveness analysis in 110 IPF subjects and baseline data comparison

In 110 IPF subjects who received pirfenidone treatment for ≥3 months, the effectiveness was analyzed by HRCT before and after pirfenidone therapy. 89 patients (80.9%) were classified to be in the stable group while 21 (19.1%) were in the progressive group, out of which 10 subjects died of acute exacerbation and 11 subjects progressed. By comparing the baseline characteristics between the above groups, it was found that there was a significant difference in BMI (*P* = 0.005), while no significant difference in age, smoking, disease duration, pirfenidone therapy time, proportion of patients with reduced dose, home oxygen therapy, FVC % predicted, TLC % predicted, or DLco % predicted (all with *P*≥0.05) (Table 3). The above-outlined factors and their effects were further analyzed in the multivariate logistic regression analysis. Only BMI (OR = 1.326, 95% CI, 1.079–1.629, *P* = 0.007) was significantly associated with the effectiveness of pirfenidone.

**Table 3:** Baseline characteristics in stable and progressive group.

	Stable disease (n = 89)	Progressive disease (n = 21)	*P* value
Total treatment time (months)	13.2±8.0	15.6±10.5	0.251
Dose reduced (1200 or 1500mg/d), n (%)	15 (16.9)	2 (9.5)	0.519
Age (years)	63±8.5	65±8.0	0.340
Male, n (%)	83 (93.3)	20 (95.2)	0.730
BMI (kg/m^2^)	24.8±2.7	22.9±3.0	0.005
Time since IPF diagnosis (years)	2.5±1.8	3.2±2.0	0.096
Smoking (including former or active smokers), n (%)	56 (62.9)	12 (57.1)	0.624
Home oxygen therapy, n (%)	34 (38.2)	10 (47.6)	0.428
FVC (% predicted)	71.7±14.7	65.6±12.7	0.083
TLC (% predicted)	66.1±10.4	61.2±10.1	0.050
DLco (% predicted)	45.9±12.3	42.6±18.3	0.315

FVC = forced vital capacity; TLC = total lung capacity; DLco = diffusion capacity of carbon monoxide.

#### Efficacy analysis by PFTs in a subgroup with 41 IPF subjects out of 110 IPF patients

Out of 110 IPF patients, only 41 subjects received PFTs at the baseline and after treatment in our hospital. In the stable group of 34 IPF subjects, a slight decline of FVC % predicted (from 68.6±13.5 to 66.7±12.8) and DLco % predicted (from 44.5±12.9 to 43.2±14.0) were noticed and they failed to meet the significant difference standard (*P* = 0.542 and *P* = 0.689 respectively). In the progressive group of 7 patients, an obvious decline was witnessed in FVC % predicted from 60.0±10.6 to 48.6±9.6, and DLco from 42.3±16.1 to 31.6±12.1, but no significant difference was found. However, there were statistically significant differences in the changes from baseline to endpoint in FVC and DLco between the stable and progressive groups (both with P<0.001). (Table 4)

**Table 4:** PFTs before and after pirfenidone therapy in stable and progressive group.

Variable Mean±SD	Stable disease (n = 34)		Progressive disease (n = 7)	
	Baseline value	Post treatment	Baseline value	Post treatment
FVC (% predicted)	68.6±13.5^a^	66.7±12.8^a^	60.0±10.6^c^	48.6±9.6^c^
DLco (% predicted)	44.5±12.9^b^	43.2±14.0^b^	42.3±16.1^d^	31.6±12.1^d^
ΔFVC (% predicted)	-2.0±5.0^e^		-11.5±6.3^e^	
ΔDLco (% predicted)	-1.3±5.4^f^		-10.7±6.9^f^	

^a^: *P* = 0.542

^b^: *P* = 0.689

^c^: *P* = 0.055

^d^: *P* = 0.185

^e^: *P*<0.001

^f^: *P*<0.001

ΔFVC, ΔDLco = the mean change in FVC or DLco from baseline to the end of follow-up

FVC = forced vital capacity; DLco = diffusion capacity of carbon monoxide.

#### Kaplan–Meier distribution for the probability of progression-free survival in 110 IPF patients

**Fig 2:**
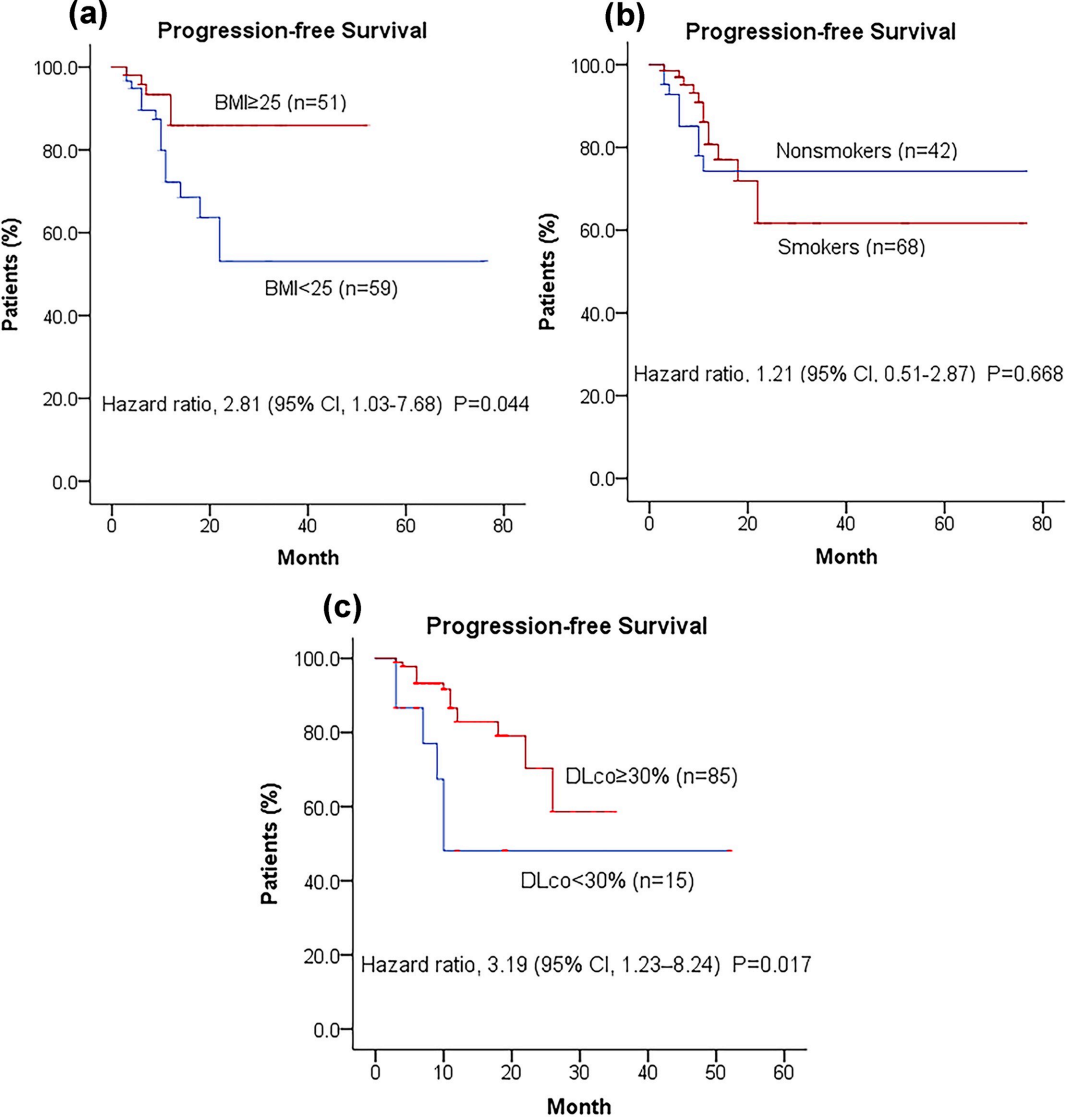
The Kaplan–Meier distribution for the probability of progression-free survival on IPF patients.

110 IPF Subjects were compared for differences in PFS with varying clinical characteristics like BMI (≥25 kg/m^2^, the overweight standard by WHO), smoking (containing ever smokers or active smokers) and lung function. Disease progression was significantly associated with BMI (<25kg/m^2^) (hazard ratio, 2.81; 95% CI, 1.03 to 7.68; *P* = 0.044), while increased BMI was associated with better prognosis (Fig 2A). Since FVC predicted of the most IPF subjects was ≥50%, we only used the cut-off value of 30% predicted for DLco (inclusion criteria cut-off values in clinical trials). Statistically significant difference was noted in PFS between IPF subjects with DLco≥30% and DLco<30% (hazard ratio 3.19; 95% CI, 1.23–8.24; *P* = 0.017). This revealed that the relative risk of death or disease progression among subjects with DLco<30% was at least 2.19 times higher than those with DLco≥30% (Fig 2C). However, no significant difference in the probability of PFS was found between smoking and non-smoking IPF patients (Fig 2B).

Binary logistic regression analysis was performed to investigate the potential correlation between decreased Dlco (<30%) and lower BMI (<25kg/m^2^) of the included subjects, and no statistically significant association was found (OR = 2.693, 95% CI, 0.801–9.057, *P* = 0.109).

### Adverse reactions and acute exacerbation/death

Adverse events that occurred during the therapy period were summarized in Table 5. Adverse events were reported in 51 subjects with IPF (43.6%), 8 with CTD-ILD (42.1%) and 16 with unclassifiable ILD (40.0%). Skin-related (20.5%, 10.5% and 17.5%) and gastrointestinal-related adverse events (35%, 31.6% and 30.0% respectively) were the most common events in the three groups. Elevations in the level of aminotransferase (the value that was 2 or more times the upper limit of the normal range) occurred in 2 patients (1.7%) in the IPF group but not in the other two groups. Death rate in the IPF group (8.5%) or CTD-ILD group (10.5%) was lower than in the unclassifiable ILD group (17.5%). The mean time to acute exacerbation or death in the three groups was from 210 to 253 days.

**Table 5:** Adverse reactions, drug discontinuation and acute exacerbation /death.

Events	IPF group (n = 117)	CTD-ILD (n = 19)	Unclassifiable ILD (n = 40)
Observation period (weeks)	52±35	50±25	41±25
Any adverse event occurred	51 (43.6)	8 (42.1)	16 (40.0)
Skin-related			
Rashes/photosensitivity/itching, n (%)	24 (20.5)	2 (10.5)	7 (17.5)
Gastrointestinal-related			
Nausea, n (%)	7 (6.0)	3 (15.8)	2 (5.0)
Vomiting, n (%)	2 (1.7)	0	1 (2.5)
Epigastric discomfort, n (%)	19 (16.2)	2 (10.5)	4 (10.0)
Acid regurgitation/heart burn, n (%)	15 (12.8)	1 (5.3)	3 (7.5)
Anorexia, n (%)	9 (7.7)	2 (10.5)	3 (7.5)
Abdominal distension, n (%)	7 (6.0)	2 (10.5)	1 (2.5)
Diarrhea, n (%)	7 (6.0)	0	0
Fatigue, n (%)	3 (2.6)	2 (10.5)	2 (5.0)
Body weight loss, n (%)	2 (1.7)	0	1 (2.5)
Blurred vision, n (%)	6 (5.1)	1 (5.3)	0
Back pain, n (%)	1 (0.9)	0	1 (2.5)
Aminotransferase elevations, n (%)	2 (1.7)	0	0
Drug discontinuation, n (%)	18 (15.4)	4 (21.1)	5 (12.5)
Time to drug discontinuation (days)	252 ± 240	340 ± 47	403 ± 308
Acute exacerbation on therapy / death, n (%)	10 (8.5)	2 (10.5)	7 (17.5)
Time to first acute exacerbation (days)	253 ± 164	210 ± 127	222 ± 146

IPF = idiopathic pulmonary fibrosis; CTD-ILD = connective tissue disease-associated ILD; ILD = interstitial lung disease.

Multivariate logistic regression analyses were performed to assess whether the clinical characteristics of the three groups were related to therapy withdrawal. The analysis of IPF group showed that age (≥70 years old) (OR = 1.392, 95% CI, 0.36–5.36, *P* = 0.630), home oxygen supply (OR = 0.46, 95% CI, 0.11–1.75, *P* = 0.254), and DLco % predicted (<30%) (OR = 1.46, 95% CI, 0.22–9.93, *P* = 0.697), had no significant association to drug withdrawal. Similar results were obtained in the CTD-ILD or unclassifiable ILD group.

### Tolerability and dose reduced

**Fig 3:**
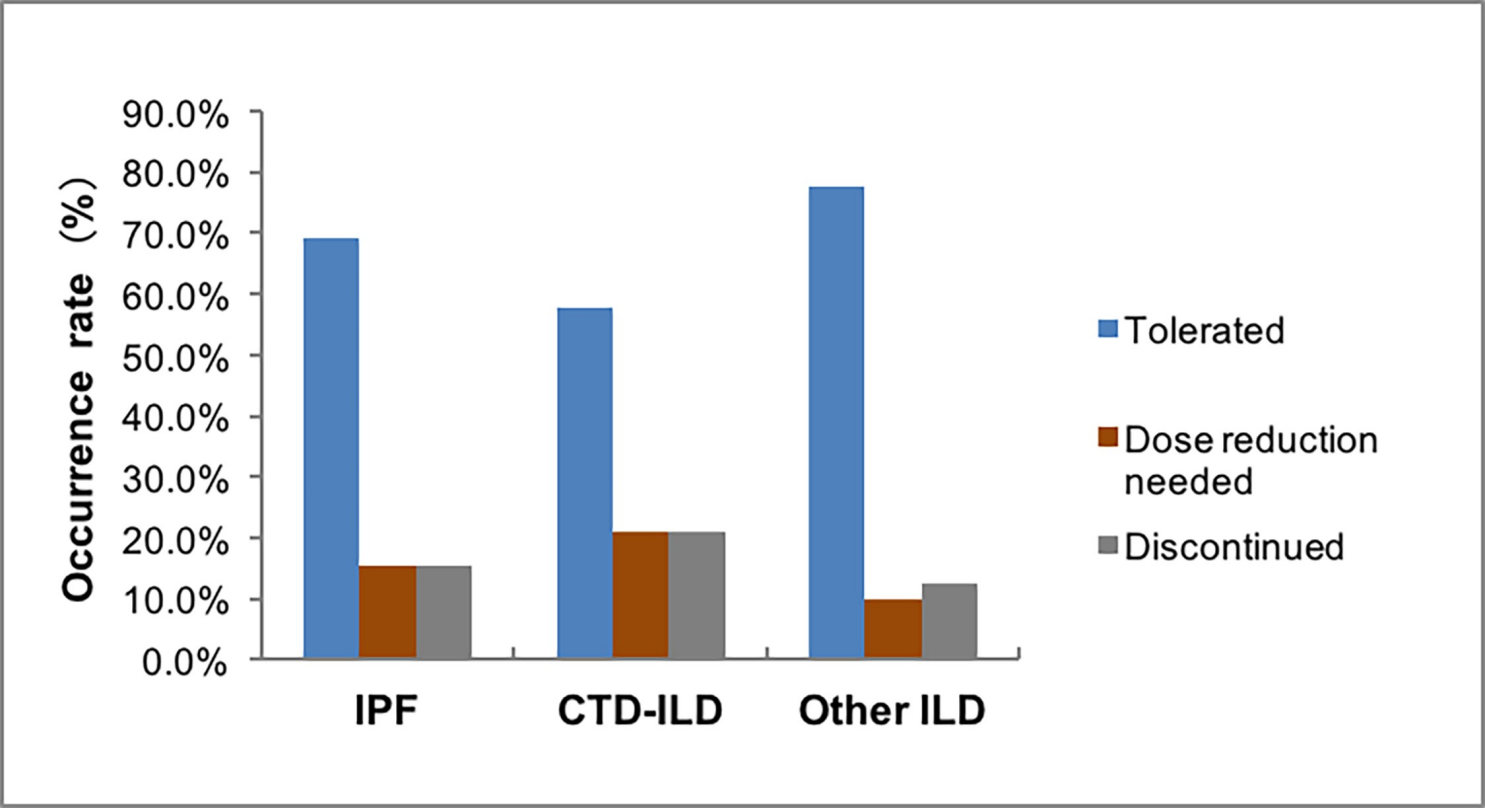
Tolerability to pirfenidone therapy.

**Fig 4:**
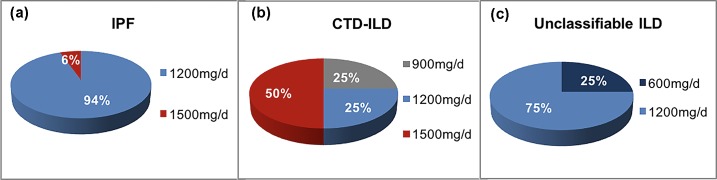
Dose reduced after adverse drug events occurred in three groups.

Most subjects in these three groups tolerated the treatment well, signifying that neither a dose reduction nor drug discontinuation was necessary. A minority of subjects (15.4%, 21.1% and 10.0% in these three groups respectively) needed to reduce dose owing to adverse events. For patients requiring a reduced dose, a larger proportion of subjects opted to 1200mg daily or 1500mg daily (Figs 3 and 4). Pirfenidone discontinuation, because of adverse events, occurred in 18 subjects with IPF (15.4%), 4 (21.1%) with CTD-ILD and 5 (12.5%) with unclassifiable ILD. Time to drug discontinuation (days) varied among the three groups, with 252 ± 240, 340 ± 47 and 403 ± 308 respectively (Table 5).

## Discussion

In this research, we mainly focused on the effectiveness of pirfenidone on IPF patients and the safety of the treatment on the subjects with various kinds of ILD. The incidence of acute exacerbation during pirfenidone therapy was 8.5% in the IPF group, 10.5% in the CTD-ILD group and 17.5% in the unclassifiable ILD group. The above data of the IPF group was similar to the former clinical trial in China (5.26% in the pirfenidone group)[20]. Approximately 80% of IPF subjects were assessed as stable by HRCT in 110 IPF patients taking pirfenidone for ≥3 months. In a subgroup of patients who received PFTs before and after pirfenidone therapy, FVC predicted and DLco predicted of IPF patients in the progressive group dropped dramatically after treatment, though without statistically significant difference compared with the baseline values (*P* = 0.055 and *P* = 0.185 respectively). Statistically significant differences were observed in the changes from baseline to the end of follow-up in FVC and DLco between stable and progressive groups, with a larger decline in the latter group (both with *P*<0.05).

The baseline BMI of IPF subjects in the progressive group was lower than that in the stable group. However, there was no significant difference in age, disease duration, baseline DLco or FVC, supplementary oxygen therapy, pirfenidone dose or treatment time between the two groups. Further evidence from the PFS analysis in Fig 2A indicated that IPF subjects with BMI<25kg/m^2^ were more prone to disease progression, acute exacerbation or death than overweight patients (BMI≥25kg/m^2^). Earlier articles held the same point that nutritional status or BMI was a predictor of prognosis for some chronic pulmonary diseases such as pulmonary tuberculosis[21], chronic obstructive pulmonary disease (COPD) [22], IPF[23,24] and cystic fibrosis[25,26]. All these studies revealed that higher BMI or good nutrition was associated with a better survival rate. The possible explanations might be as follows. (1) It was reported in one former research[27] that BMI influenced the natural history of asthma and had different impacts on the responsiveness to asthma therapy (inhaled corticosteroid and leukotriene antagonist). Therefore, it was inferred that BMI might influence the disease’s course and the response to pharmacologic treatment[23]. (2) Higher BMI might mean improved nutritional status[23]. Nevertheless malnutrition status was related to thymic atrophy and reduced T-lymphocyte function, thereby increasing infection risk[28]. However, the specific mechanism by which BMI affects the prognosis of IPF is still unclear and more related studies are still required to investigate their association and mechanism.

The CAPACITY and ASCEND trials recruited subjects with predicted FVC of at least 50% and DLco of at least 35% or 30%. Most patients of this real-world study had almost the same requirement in predicted FVC as clinical trials (≥50%), whereas some with DLco<30%. Kaplan–Meier distribution for the probability of progression-free survival showed that IPF patients with severe baseline PFTs (DLco<30%) were 3 times more likely to develop disease progression or acute exacerbation or death than the IPF subjects with DLco≥30%. The possible explanation for this is patients with worse physiology are likely to present a later stage of the natural history of IPF and they have lower survival chance than those with preserved physiology[29]. At the same time, we should note that advanced IPF patients with lower DLco have a higher risk of developing pulmonary hypertension (PH)[30,31] and that PH has been reported to be associated with poor prognosis of IPF[31,32]. So PH may contribute to the worse outcome in those with lower Dlco. However, there is no routine screening for PH in IPF patients in the real-world clinical practice. Therefore, the relationship between IPF prognosis and DLco needs to be further confirmed in the future studies considering the factor of PH. In terms of pirfenidone therapy in advanced IPF, pirfenidone demonstrated clinically relevant benefits and well tolerability in several recent studies[33–35]. The former research had demonstrated that smoking was a potential risk factor for the development of IPF (OR: 1.6–2.9)[36]. Furthermore, one research by Antoniou et al[37] found that survival was higher in nonsmokers than in former smokers or the combined group of former and active smokers. The possible explanation for this might be that cigarette smoking induced IPF combined with emphysema, leading to further impaired physiological function[38]. However, PFS analysis about smoking status in IPF patients demonstrated that smoking was not statistically associated with the poor prognosis of IPF patients (Fig 2B). Maybe it is difficult to draw a conclusion based on the results of the aforementioned studies alone and further research is required to analyze the association of smoking status with IPF prognosis.

Only patients diagnosed with IPF were recruited in CAPACITY and ASCEND clinical trials. However, our real-world study included patients diagnosed with IPF, CTD-ILD or unclassifiable ILD. We had found that the incidence of adverse events in the above three kinds of ILD was almost the same, around 40%, which was lower than the previous study[39]. And the most common adverse events of these groups were skin-associated (rashes/photosensitivity/itching) and gastrointestinal, with epigastric discomfort reported most frequently in the IPF or unclassifiable ILD group and nausea in the CTD-ILD group. The rates of adverse events in our study were lower than the pooled clinical trial data[40]. These events were generally recoverable and responsive to dose reduction or other measures like taking pirfenidone with food and avoiding sun exposure. A majority of patients in three groups including IPF (69.2%), CTD-ILD (57.9%) and unclassifiable ILD (77.5%) had sufficient tolerance to pirfenidone. Adverse events led to discontinuation of pirfenidone treatment in 18 patients in the IPF group (15.4%), 4 patients in the CTD-ILD group (21.1%) and 5 patients in the unclassifiable ILD group (12.5%). The rates of drug discontinuation in this research was similar to the clinical trials[9, 10] and two earlier real-world observational studies[39, 41].

There were several limitations to this study. First, this was a retrospective study and nonstandardization of follow-up inevitably occurs, possibly giving rise to missed data like adverse events. However, we have tried to minimize the loss of data through a thorough investigation of patients’ electronic and paper medical records. Additionally, this was a single-arm study which lacked a control group, that might cause confounding in the estimates. Second, a majority of patients in the real world did not have regular lung function tests and most of them had these tests in different hospitals, leading to the failure to assess effectiveness by PFTs according to one standardization. Third, patients in our real world had some comorbidities or concurrent medication use, which might have an impact on the effectiveness and tolerability of pirfenidone. Fourth, due to the limited size sample in the two groups of patients with other kinds of ILD in addition to IPF, we did not assess their effectiveness in this study, which we would study in the future.

## Conclusion

This retrospective single-arm research spanning four years and two months with pirfenidone on various kinds of ILD patients demonstrated well safety profile and similar discontinuation rates to previously published data[9, 10, 39, 41]. Our data provided further ongoing evidence regarding the safety and tolerability of pirfenidone therapy for a broader cohort of patients in addition to IPF patients in the real world. In this study, more benefits were witnessed in IPF patients with higher BMI or mild-to-moderate disease. However, the effectiveness of pirfenidone treatment on patients with CTD-ILD and other ILDs is still needed to be demonstrated.

## Supporting information

S1 Dataset Dataset of patients with pulmonary fibrosis. (XLSX)
